# Interrelationship of Gut Microbiota, Obesity, Body Composition and Insulin Resistance in Asians with Type 2 Diabetes Mellitus

**DOI:** 10.3390/jpm12040617

**Published:** 2022-04-11

**Authors:** Che-Sheng Pai, Cheng-Yuan Wang, Wei-Wen Hung, Wei-Chun Hung, Hui-Ju Tsai, Chen-Chia Chang, Shang-Jyh Hwang, Chia-Yen Dai, Wen-Yu Ho, Yi-Chun Tsai

**Affiliations:** 1 Department of Internal Medicine, Division of Endocrinology and Metabolism, Kaohsiung Medical University Hospital, Kaohsiung Medical University, Kaohsiung 807, Taiwan; whitejason31.jw@gmail.com (C.-S.P.); hung4488@ms57.hinet.net (W.-W.H.); sjhwang730136@gmail.com (S.-J.H.); 2 Department of Internal Medicine, Division of General Medicine, Kaohsiung Medical University, Kaohsiung Medical University Hospital, Kaohsiung 807, Taiwan; ayuan73@gmail.com; 3 Department of Microbiology and Immunology, College of Medicine, Kaohsiung Medical University, Kaohsiung 807, Taiwan; wchung@kmu.edu.tw (W.-C.H.); wacsun@gmail.com (C.-C.C.); 4 Department of Family Medicine, Kaohsiung Municipal Ta-Tung Hospital, Kaohsiung Medical University, Kaohsiung 807, Taiwan; bankin_0920@yahoo.com.tw; 5 Department of Internal Medicine, Division of Nephrology, Kaohsiung Medical University Hospital, Kaohsiung Medical University, Kaohsiung 807, Taiwan; 6 School of Medicine, College of Medicine, Kaohsiung Medical University, Kaohsiung 807, Taiwan; daichiayen@gmail.com; 7 Department of Internal Medicine, Division of Hepatobiliary, Kaohsiung Medical University Hospital, Kaohsiung Medical University, Kaohsiung 807, Taiwan; 8 Liquid Biopsy and Cohort Research Center, Kaohsiung Medical University, Kaohsiung 807, Taiwan

**Keywords:** gut microbiota, body composition, obesity, type 2 diabetes mellitus, insulin resistance, *A. muciniphila*

## Abstract

Metabolic syndrome (MS) has been an important health issue in the world, and insulin resistance (IR) is one of the characteristics of MS, increasing the risk for the onset and poor prognosis of type 2 diabetes mellitus (T2D). However, the interactional effect of obesity or abnormal body composition on the correlation between gut microbiota and IR in T2D patients is not well-explored. This cross-sectional study used a body composition monitor to evaluate lean tissue mass and fat tissue mass. IR was calculated using homeostatic model assessment-insulin resistance (HOMA-IR). Eight pairs of 16S rRNA gene primers specific to *Firmicutes*, *Bacteroidetes*, *Clostridium leptum* group, *Faecalibacterium* *prausnitzii*, *B acteroides*, *Bifidobacterium*, *Akkermansia muciniphila*, and *Escherichia coli* were utilized to measure their abundance by qPCR. One hundred and fifty-four T2D patients were enrolled and stratified by the median HOMA-IR (2.5) and body mass index (BMI) of 25 kg/m^2^. A lower abundance of *A. muciniphila* was found in T2D patients with high HOMA-IR and BMI respectively. HOMA-IR and BMI had a synergistic effect on the reduction of the abundance of *A. muciniphila*. After adjusting metabolic factors, the low abundance of *A. muciniphila* significantly increased the risk for greater severity of IR. Furthermore, the negative correlation between *A. muciniphila* and IR was only found in T2D patients with high lean tissue. In conclusion, decreased abundance of fecal *A. muciniphila* enhanced the severity of IR in Asians with T2D, especially those having lean mass, and this significant relationship was independent of obesity.

## 1. Introduction

Metabolic syndrome became a worldwide health issue for decades, both in developed and underdeveloped countries [1,2,3]. The syndrome is characterized by a series of comorbidities, including hypertension, central obesity, glucose intolerance, insulin resistance (IR), and dyslipidemia, that increase the individual’s risk of type 2 diabetes mellitus (T2D) [4]. Among the above features, IR causes the inability of glucose transport and utilization by cells, leading to hyperinsulinemia and other manifestations of metabolic syndrome [5]. Thus, IR is directly linked to the development and poor prognosis of T2D. On the other hand, obesity is known to be a feature of metabolic syndrome and may trigger T2D with IR through higher levels of non-esterified fatty acids, glycerol, and pro-inflammatory cytokines released by adipose tissue. In addition, lean tissue mass (LTM), as free-fat mass, has the principal role in resulting in IR [6]. LTM is the target tissue of insulin and can produce myokines [6]. Maintenance and increase of the LTM might be useful for the improvement of IR. The impact of the interaction of obesity or variation of body composition in IR and the further onset or progression of T2D is important and complicated.

Numerous approaches are undergoing to further understand the etiology of IR and prevent its pathological process. Among them, gut microbiota plays an important role in T2D with IR [7,8,9,10]. Gut microbiota conducts a complicated system to modulate intestinal barrier and metabolic endotoxin secretion [11]. Lipopolysaccharide (LPS) secreted from intestinal flora induces chronic subclinical inflammation and obesity through activation of Toll-like receptor 4 (TLR4), leading to IR [12]. More than 90% of gut microbiota could be divided into two main phyla *Firmicutes* and *Bacteroidetes* [13]. Individuals with obesity or T2D patients were found to have reduced *Firmicutes/Bacteroidetes* ratio, which regulates the pathophysiologic process of metabolic disorders [13]. *Bacteroidetes* are gram-negative bacteria and contain LPS, which could activate TLR4, inducing inflammation and IR [13]. On the other hand, gut microbiota could degrade nondigestible carbohydrates to short-chain fatty acids (SCFA) including acetate, butyrate, and propionate. SCFA serves as an energy source to the intestine cells to maintain epithelial barrier function [12], and low levels of SCFA have been related to increased risk for IR and inflammation [14]. Accumulating evidence indicates that individuals with prediabetes and T2D had a lower abundance of butyrate-producing gut microbiota, such as *Faecalibacterium prausnitzii* (*F. prausnitzii*), genus *Bifidobacterium*, and *Akkermansia muciniphila* (*A. muciniphila*) [14,15,16,17]. *F. prausnitzii*, being part of the *Clostridium leptum* (*C. leptum*) *group*, is the most abundant bacterium in the intestinal microbiota of healthy individuals [15]. Decreased abundance of *A. muciniphila* could increase intestinal permeability, promoting metabolic endotoxin penetrating the bloodstream and contributing to metabolic syndrome [18]. In addition, lower concentrations of genus *Bacteroides*, belonging to phylum *Bacteriodetes*, and a high abundance of *Escherichia coli* (*E. coli*) were also found in individuals with metabolic syndrome, including T2D and non-alcohol fatty liver disease (NAFLD) [19,20]. Nevertheless, the interactional effect of obesity and abnormal body composition on the correlation between gut microbiota and IR in T2D patients is not well-explored. Therefore, the aim of this study was to explore the relationship between the severity of IR and the abundances of targeted fecal bacterial species, including phyla *Firmicutes* and *Bacteriodetes*, *C. leptum group*, *F. prausnitzii*, genera *Bacteroides* and *Bifidobacterium*, *A. muciniphila*, and *E. coli* in T2D patients stratified by obesity or body composition.

## 2. Materials and Methods

### 2.1. Study Participants

This observational study enrolled 154 T2D patients ages 18-years or above at a tertiary hospital in Southern Taiwan from October 2016 to December 2017. All study subjects had received the principles of diet therapy and the T2D education program. Enrolled patients could use any hypoglycemic or lowering-lipid drugs; however, patients who had used antibiotics and probiotic or prebiotic products before enrollment in this study were excluded. The study protocol was approved by the Institutional Review Board of Kaohsiung Medical University Hospital (KMUHIRB-G(II)-20160021). Informed consent was obtained in written form from all the patients, and all clinical investigations were conducted according to the principles expressed in the Declaration of Helsinki.

### 2.2. Sample and Clinical Data Collection

T2D was defined as a history of diabetes or the use of anti-diabetic agents, and blood glucose values using American Diabetes Association criteria. Demographics, such as a history of cigarette smoking and alcohol drinking, and clinical data were obtained from interviews with the patients and medical records at enrollment. Hypertension was defined as a history of hypertension or the use of antihypertensive drugs. Hyperlipidemia was defined as a history of hyperlipidemia or the use of statin or fibrate. Gout was defined as history gout or the use of colchicine or lowering-uric-acid agents. Information on the use of medications including anti-diabetic agents and statins at enrollment was obtained from medical records. We recorded usual diet habits in these patients using a simple questionnaire. Body mass index (BMI) was calculated as body weight (kg) divided by body height (m) squared. Obesity was defined as a BMI of 25 kg/m^2^ or above in the Asian population as an Asia-Pacific Perspective recommendation [21]. Homeostatic model assessment-insulin resistance (HOMA-IR) was calculated as glucose (mg/dL) × insulin (mIU/L) divided by 405. The patients were asked to fast for 12 h before blood sample collection for biochemistry studies.

### 2.3. Stool Sample Collection and Microbial DNA Extraction

Fecal samples were collected on the same day of blood collection as in our previous studies [11,22]. In brief, the fecal samples were collected and stored at −80 °C for up to three days before processing. A stool DNA Extraction kit (Topgen Biotechnology Co., Ltd., Kaohsiung, Taiwan) was used to extract bacterial DNA. The fecal samples weighted to 50 to 100 mg were supplemented using a preceding bead beating (45 s; 3450 oscillations/min). The subsequent steps of DNA extraction were performed according to the manufacturer’s protocol. DNA concentration and quality were assessed using the Colibri Microvolume spectrophotometer (Titertek Berthold, Pforzheim, Germany). Extracted DNA samples were immediately stored at −20 °C before use.

### 2.4. Real-Time Quantitative Polymerase Chain Reaction (qPCR)

Eight pairs of 16S rRNA gene primers specific to *Bacteroidetes*, *Firmicutes*, *C. leptum* group, *F. prausnitzii*, *Bacteroides*, *Bifidobacterium*, *A. muciniphila*, and *E. coli* in feces were measured by real-time qPCR in StepOnePlus Real-Time PCR system (Thermo Fisher Scientific, Waltham, MA, USA) as in the previous study (Table S1) [11,22]. Standard curves were constructed with a 10-fold dilution series of the 16S rDNA gene fragment amplified from the reference strains that was cloned in a T&A^TM^ Cloning Vector (Yeastern Biotech, Co., Ltd., Taipei, Taiwan). Each reaction mixture with a total volume of 10 μL was composed of 0.25 μL of each 10 μM primers, 5 μL AceQ qPCR SYBR Green Master Mix (Vazyme Biotech Co., Piscataway, NJ, USA), 1 μL of sample DNA, and 3.5 μL sterilized ultra-pure water. Real-time PCR was carried out by the following cycle conditions: an initial holding at 95 °C for 30 s, followed by 40 cycles of denaturation at 95 °C for 3 s, then annealing/elongation at 60 °C for 40 s. Melting curve analysis was performed after amplification to determine the specificity, and the presented data are the mean values of duplicate qPCR analysis.

### 2.5. Measurement of Body Composition

Body composition including lean tissue and fat tissue was measured once by a bioimpedance spectroscopy method, Body Composition Monitor (BCM, Fresenius Medical Care, Bad Homburg, Germany) at enrollment. The BCM, which has been validated against gold-standard methods in the general population, measures impedance spectroscopy at 50 different frequencies from 5 kHz to 1 MHz [23,24]. Patients were in the recumbent position for at least 5 min, and then electrodes were attached to one hand and one foot on the ipsilateral side. Only the parameters in which the quality of the measurement was 95% or above were included in the analysis. BCM provides information on normohydrated lean tissue, normohydrated adipose tissue, and extracellular fluid overload in the whole body based on the difference in impedance in each tissue [25]. Normohydrated lean tissue and normohydrated adipose tissue were presented as lean tissue index (LTI) and fat tissue index (FTI) respectively.

### 2.6. Statistical Analysis

The baseline characteristics of the patients were stratified by the median of HOMA-IR and BMI of 25 kg/m^2^ or above. Continuous variables were expressed as mean ± SD or median (25th, 75th percentile), as appropriate, and categorical variables were expressed as percentages. Continuous variables with skewed distribution were log-transformed to approximate normal distribution. The significance of differences in continuous variables between groups was tested using ANOVA or the Kruskal-Wallis H analysis, as appropriate. Differences in the distribution of categorical variables were tested using the chi-square test. Multivariate forward logistic regression models were used to evaluate the association between the microbiota and the severity of HOMA-IR. All the variables in Table 1 were tested by univariate analysis and those variables with *p*-value < 0.05, age, and sex were selected in multivariate analysis. Statistical analyses were conducted using SPSS version 22.0 for Windows (SPSS Inc., Chicago, IL, USA) and the graphs were made by GraphPad Prism 9.0 (GraphPad Software Inc., San Diego, CA, USA). Statistical significance was set at a two-sided *p*-value of < 0.05.

## 3. Results

### 3.1. Characteristics of Entire Cohort

The comparison of clinical characteristics between groups based on the median of HOMA-IR (2.5) and BMI of 25 kg/m^2^ is shown in Table 1. Of 154 T2D patients, the mean age was 63.1 ± 9.7 years, 58.4% were male, the diabetic duration was 9.2 ± 8.4, and the mean BMI was 26.7 ± 3.9 kg/m^2^. The prevalence of hypertension, gout, and hyperlipidemia was 62.3%, 10.7%, and 83.0% respectively. T2D patients with high HOMA-IR and BMI ≥ 25 kg/m^2^ had the youngest age, the highest proportion of hypertension, and the highest FTI among the four groups. There was no difference in the proportion of smoking, alcohol, diet habit, or statin usage among the four groups. The highest cholesterol, triglyceride, and glycated hemoglobin levels were found in T2D patients with high HOMA-IR and BMI ≥ 25 kg/m^2^ among the four groups. T2D patients with high HOMA-IR had lower high-density lipoprotein levels compared to those with low HOMA-IR.

**Table 1:** The characteristics of study participants stratified by the median of HOMA-IR and BMI of 25 kg/m^2^.

	Entire Cohort (*n* = 154)	HOMR-IR <Median & BMI < 25 (*n* = 35)	HOMR-IR <Median & BMI ≥ 25 (*n* = 40)	HOMR-IR ≥Median & BMI < 25 (*n* = 22)	HOMR-IR≥Median & BMI ≥ 25 (*n* = 57)	*p*-Value
Age, year	63.1 ± 9.7	64.9 ± 9.7	65.3 ± 9.0	66.3 ± 6.8	59.1 ± 10.0	0.001
Sex (male), %	58.4	74.3	65.0	40.9	50.9	0.04
Smoke, %	30.5	37.1	30.0	27.3	28.1	0.80
Alcohol, %	22.7	37.1	20.0	22.7	15.8	0.11
Hypertension, %	62.3	36.1	70.0	54.5	75.4	0.001
Gout, %	10.7	5.6	12.5	13.6	11.5	0.71
Hyperlipidemia, %	83.0	80.6	82.5	72.7	88.5	0.37
DM duration, year	9.2 ± 8.4	9.2 ± 9.3	8.3 ± 9.7	9.5 ± 6.1	9.5 ± 7.7	0.90
Body Mass Index, kg/m^2^	26.7 ± 3.9	22.9 ± 1.5	27.8 ± 2.3	23.1 ± 1.3	29.6 ± 3.5	<0.001
Diet habit, %						0.45
Protein more than fiber	18.1	14.3	15.8	9.1	25.9	
Fiber more than protein	26.2	34.3	21.1	31.8	22.2	
Fiber equal to protein	55.7	51.4	63.2	59.1	51.9	
Medication						
Sulfonylurea (yes vs. no)	45.9	44.4	30.0	45.5	57.4	0.06
DPP4 inhibitor (yes vs. no)	73.0	58.3	75.0	81.8	77.0	0.15
Metformin (yes vs. no)	85.5	91.7	75.0	95.5	85.5	0.09
Actos (yes vs. no)	3.8	5.6	7.5	0.0	1.6	0.32
Insulin (yes vs. no)	19.5	2.8	5.0	31.8	34.4	<0.001
Statin (yes vs. no)	61.6	55.6	67.5	50.0	65.6	0.42
Body composition						
Lean mass index, kg/m^2^	12.0 ± 2.1	11.9 ± 1.9	12.2 ± 2.0	10.8 ± 1.8	12.3 ± 2.2	0.07
Fat mass index, kg/m^2^	14.4 ± 4.1	10.4 ± 2.2	15.3 ± 3.0	12.2 ± 1.9	17.5 ± 3.8	<0.001
Laboratory parameters						
HOMA-IR	2.5 (1.6, 4.5)	1.6 (1.1, 2.0)	1.6 (1.2, 2.2)	3.6 (2.9, 5.7)	5.2 (3.3, 8.7)	<0.001
Cr, mg/dL	1.0 ± 0.5	1.0 ± 0.4	1.1 ± 0.6	0.9 ± 0.3	1.1 ± 0.5	0.27
Hemoglobin, g/dL	13.5 ± 1.8	13.5 ± 1.8	13.0 ± 1.9	13.4 ± 1.6	13.9 ± 1.7	0.12
Albumin, g/dL	4.6± 0.2	4.6 ± 0.2	4.6 ± 0.2	4.6 ± 0.2	4.5 ± 0.2	0.11
Uric acid, mg/dL	6.0 ± 1.6	5.8 ± 1.7	6.3 ± 1.6	5.8 ± 1.4	6.0 ± 1.6	0.42
Cholesterol, mg/dL	166.5 ± 42.6	157.7 ± 24.8	153.6 ± 39.1	165.4 ± 41.0	181.5 ± 50.0	0.006
Triglyceride, mg/dL	129 (90, 184)	99 (68, 129)	112 (86, 164)	122 (85, 186)	162 (129, 258)	<0.001
HDL, mg/dL	44.5 ± 22.5	45.8 ± 11.0	45.8 ± 27.9	42.1 ± 12.2	43.7 ± 26.5	0.02
LDL, mg/dL	92.5 ± 32.5	89.8 ± 23.1	86.4 ± 34.4	94.8 ± 32.0	97.5 ± 36.1	0.43
Glycated hemoglobin, %	7.0 (6.4, 8.0)	6.8 (6.2, 7.1)	6.5 (6.1, 7.0)	7.2 (6.8, 8.6)	7.8 (6.9, 9.0)	<0.001

Abbreviations: HOMA-IR, homeostatic model assessment-insulin resistance; BMI, body mass index; DPP4, Dipeptidyl peptidase 4; Cr, creatinine; HDL, high-density lipoprotein; LDL, low density-lipoprotein.

### 3.2. The Distribution of Gut Microbiota in T2D Patients Stratified by HOMA-IR and BMI

The levels of phyla *Firmicutes* and *Bacterodietes*, *C. leptum group*, *genera Bacteroides* and *Bifidobacterium*, *A. muciniphila*, *F. prausnitzii*, and *E. coli* were examined using qPCR in the study subjects. In T2D patients with low HOMA-IR, those with a BMI ≥ 25 kg/m^2^ had decreased abundance of *A. muciniphila* compared to those with a BMI < 25 kg/m^2^. In T2D patients with a BMI < 25 kg/m^2^, a lower abundance of *A. muciniphila* was found in those with high HOMA-IR than those with low HOMA-IR. T2D patients with high HOMA-IR and a BMI ≥ 25 kg/m^2^ had the lowest abundance of *A. muciniphila* among the four groups. There was no difference in the abundance of phyla *Firmicutes* and *Bacterodietes*, *C. leptum group*, genera *Bacteroides* and *Bifidobacterium*, *F. prausnitzii*, and *E. coli* among four groups (Table 2).

We also examined the impact of medications on the distribution of gut microbiota in T2D patients and found a significant difference in the abundance of *F. prausnitzii* and *E.coli* in T2D patients with and without using sulfonylurea (Table S2). T2D patients using insulin had a lower abundance of phyla *Firmicutes*, *F. prausnitzii*, and genus *Bifidobacterium* compared to those without using insulin. There was a significant difference in *A. muciniphila* in T2D patients with and without using metformin. T2D patients using a DPP-4 inhibitor had a higher abundance of *E.coli* than those without using a DPP-4 inhibitor. There was no difference in the abundance of microbiota between T2D patients with and without lowering lipid agents.

### 3.3. Gut Microbiota and the Severity of HOMA-IR

To investigate the determinants of the severity of insulin resistance, logistic analysis was used. In univariate analysis, young age, female, high BMI, sulfonylurea use, insulin use, high cholesterol, triglyceride, and glycated hemoglobin levels, and *A. muciniphila* were significantly associated with increased risk for high HOMA-IR in T2D patients (Table 3). Further multivariate forward analysis adjusting for age, sex, BMI, medications, and serum cholesterol, triglyceride and glycated hemoglobin levels, showed that T2D patients with a low abundance of *A. muciniphila* (odds ratio (OR): 0.80, 95% confidence index (CI): 0.66–0.99) had increased risk for high HOMA-IR.

**Figure 1:**
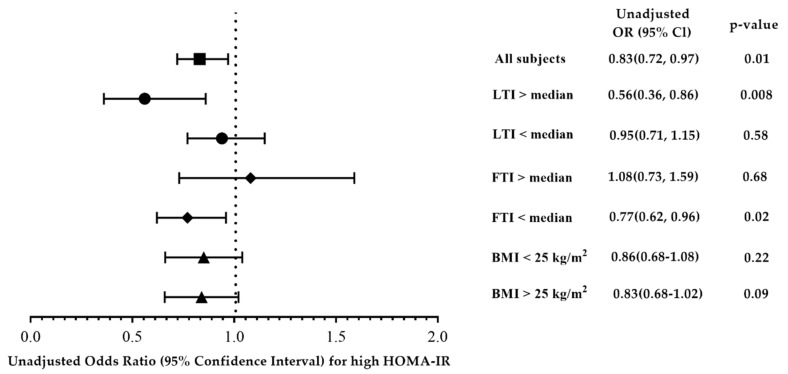
Adjusted odds ratios (ORs) of Log-formed *A. muciniphila* and high HOMA-IR (>median) in T2D patients stratified by lean tissue index (LTI), fat tissue index (FTI), and body mass index (BMI) cut at 25 kg/m^2^. Ratios were adjusted for age, sex, BMI, cholesterol, log-formed triglyceride, and glycated hemoglobin.

In order to investigate the effect of body composition on the correlation between the abundance of *A. muciniphila* and high HOMA-IR, we stratified the study subjects by LTI and FTI (Figure 1), and the results revealed a significant negative relationship between *A. muciniphila* and high HOMA-IR in T2D patients with high LTI or low FTI, not in those with low LTI and high FTI.

## 4. Discussion

This study investigated the interactional effect of obesity and distribution of body composition on the relationship between gut microbiota and IR in T2D patients. We found a lower abundance of *A. muciniphila* in T2D patients with high HOMA-IR and BMI respectively. In addition, HOMA-IR and BMI had a synergistic effect on the reduction of the abundance of *A. muciniphila*, meaning that IR and obesity affect *A. muciniphila* expression in the T2D group. After adjusting metabolic factors, including age, sex, BMI, cholesterol, triglyceride, and glycated hemoglobin, a low abundance of *A. muciniphila* significantly increased the risk for greater severity of IR in T2D patients. Furthermore, the negative correlation between *A. muciniphila* and IR was only found in T2D patients with high lean tissue, not in those with high-fat tissue. This study provided the meticulous interaction among gut microbiota, HOMR, obesity, and body composition in T2D patients.

*A. muciniphila*, a gram-negative bacterium belonging to *Verrucomicrobia* phylum, was first identified in human feces in 2004 and accounted for about 3–5% of gut microbiota in healthy adults [26]. *A. muciniphila* drew much attention in recent years since it was found reversely associated with human health and metabolic syndrome, including T2D and IR [27]. The mechanism of *A. muciniphila* as the potential therapeutic agent has not been fully understood. *A. muciniphila* has been identified as a mucin-degrading bacteria that resides in the mucus layer and could enhance mucus thickness and intestinal barrier function, further triggering both host metabolic and immune responses and stimulating beneficial mucosal microbial networks [28]. Prior studies have reported that a supplement of *A. muciniphila* could improve glucose homeostasis and reduce fasting sugar in a mouse model of Alzheimer’s disease and a high-fat diet [26,29]. Supplements of *A. muciniphila* in obese and T2D mice not only restored mucus thickness but also reduced serum LPS, leading to the repair of the intestinal membrane permeability in the in vivo study [30]. The integrity of mucus and intestinal membrane are very important to protect the host from toxin and pathogen invasion. In our study, we found that a low abundance of *A. muciniphila* in feces was significantly associated with an increased risk for IR in T2D patients. However, the interventional studies on *A. muciniphila* are limited to in vivo experiments, and limited studies with relatively small numbers have explored its safety and efficacy in humans. A further large clinical study is necessary to examine the therapeutic effect of *A. muciniphila* in patients with metabolic syndrome, including T2D.

A previous study has found the abundance of *A. muciniphila* was inversely correlated with body weight in mice and humans [31]. *A. muciniphila* modulates obesity by regulating metabolism and energy hemostasis and improving insulin sensitivity and glucose hemostasis [32]. Oral supplements of *A. muciniphila* was found to protect mice from fat-diet-related obesity [33]. However, the relationship between body composition, IR, and the abundance of *A. muciniphila* was not well elucidated in T2D patients. Our study further indicated a significant negative relationship between *A. muciniphila* and IR in T2D patients having high lean mass, not in those having high-fat mass. Conversely, this relationship was not significant in T2D patients with or without obesity. Whether *A. muciniphila* has a greater impact on IR in T2D patients with high lean mass will need to be examined in a future study.

Obesity is a key feature and risk factor of metabolic syndrome and its complications including T2D. Adipocyte hypertrophy is associated with chronic inflammation and contributes to IR [34]. BMI is usually used to assess obesity. However, BMI could not distinguish fat from muscle. Since obesity is featured with dysfunctional fat tissue, body composition measurement might be more precise to evaluate real obesity and differentiate the distribution of body fat mass and LTM [35]. LTM is metabolically involved in active processes including resting energy expenditure, glucose uptake, and myokine secretion [36]. In addition, higher levels of fat mass have been positively associated with the occurrence of metabolic syndrome independent of BMI [37]. Some reports indicated that more than half of Americans have a normal BMI but a high body fat percentage, known as normal weight obesity [38]. Thus, body composition might provide detailed information for physical clinicians to diagnose real obesity.

This study had some limitations. One of our limitations was a relatively limited number of study subjects. Nevertheless, we still found a strong correlation between gut microbiota and IR in T2D patients. In addition, the cross-sectional study design did not demonstrate the cause-effect relationship between gut microbiota and IR, and might even lead to random results. A future longitudinal study of gut microbiota and IR is needed to confirm our novel findings. Finally, this study only measured eight targeted gut microbiota by real-time qPCR instead of by 16S rRNA sequencing, which could present the whole microbiome signature pertaining to IR. However, 16S rRNA sequencing only provides a relative percentage of gut microbiota; otherwise, real-time qPCR is a cost-effective and time-saving tool to measure the absolute quantity for useful applications in clinical practice.

In conclusion, this study demonstrated a significant negative relationship between *A. muciniphila* and IR in T2D patients with high lean mass. Our findings emphasized the importance of body composition in the relationship between gut microbiota and IR. A future interventional study using A. muciniphila and other possible probiotics as a therapeutic agent for T2D patients might consider the interactional effect of body composition.

## Figures and Tables

**Table 2:** The microbiome distribution of study participants stratified by the median of HOMA-IR and BMI of 25 kg/m^2^.

	Entire Cohort (*n* = 154)	HOMR-IR <Median & BMI < 25 (*n* = 35)	HOMR-IR <Median & BMI ≥ 25 (*n* = 40)	HOMR-IR ≥Median & BMI < 25 (*n* = 22)	HOMR-IR≥Median & BMI ≥ 25 (*n* = 57)	*p*-Value
*Firmicutes*, copies × 10^9^/g	4.4 (2.3, 7.2)	4.7 (2.6, 8.8)	5.0 (2.3, 6.6)	3.7 (2.6, 8.5)	3.8 (1.9, 7.2)	0.69
*Bacteroidetes*, copies × 10^9^/g	8.9 (3.8, 17.2)	14.6 (4.6, 19.9)	9.0 (3.3, 17.2)	8.8 (4.5, 18.4)	8.3 (3.1, 13.6)	0.33
*Firmicutes/Bacteroidetes*	0.5 (0.2, 1.1)	0.5 (0.3, 1.1)	0.6 (0.2, 1.2)	0.5 (0.1, 1.0)	0.5 (0.3, 1.3)	0.92
*C. leptum group*, copies × 10^8^/g	5.9 (2.2, 11.6)	4.8 (1.9, 12.2)	6.9 (2.2, 11.8)	6.4 (3.0, 16.5)	5.0 (1.7, 9.4)	0.54
*F. prausnitzii*, copies × 10^7^/g	11.0 (2.0, 28.3)	11.6 (1.7, 28.6)	1.5 (3.9, 28.5)	9.0 (1.3, 30.6)	7.5 (1.7, 26.9)	0.67
*Bacteroides*, copies × 10^9^/g	1.6 (0.8, 3.6)	1.8 (1.1, 2.8)	2.3 (0.8, 4.4)	1.6 (0.8, 4.2)	1.4 (0.9, 3.6)	0.89
*Bifidobacterium*, copies × 10^6^/g	3.6 (0.3, 13.8)	4.3 (0.4, 15.4)	4.2 (0.1, 10.4)	5.5 (0.8, 31.9)	1.7 (0.2, 12.1)	0.37
*A. muciniphila*, copies × 10^4^/g	0.8 (0.2, 410.0)	3.4 (0.4, 767.2)	2.0 (0.1, 132.0)	1.3 (0.4, 29.0)	0.4 (0.1, 22.1)	0.01
*E. coli*, copies × 10^8^/g	1.2 (0.3, 5.9)	1.1 (0.4, 4.5)	1.2 (0.1, 9.7)	1.8 (0.8, 22.3)	1.2 (0.3, 5.9)	0.28

Abbreviations: HOMA-IR, homeostatic model assessment-insulin resistance; BMI, body mass index.

**Table 3:** Logistic regression of determinants of high HOMA-IR (≥median).

High HOMA-IR	Crude OR (95%Cl)	*p*-Value	Adjusted OR (95%Cl) (Forward)	*p*-Value
Clinical data				
Age, year	0.96 (0.92, 0.99)	0.01	-	-
Sex (male vs. female)	0.41 (0.21, 0.79)	0.008	0.29 (0.12–0.70)	0.006
Body mass index, kg/m^2^	1.19 (1.07, 1.30)	0.001	1.12 (0.99–1.26)	0.05
Smoke (yes vs. no)	0.77 (0.38, 1.53)	0.46	-	-
Alcohol (yes vs. no)	0.55 (0.25, 1.19)	0.13	-	-
Diet habit, %				
Fiber more than protein	0.88 (0.42, 1.83)	0.73	-	-
Protein more than fiber	1.50 (0.65, 3.50)	0.34	-	-
Protein equal fiber	0.86 (0.45, 1.65)	0.66	-	-
Sulfonylurea (yes vs. no)	2.03 (1.07–3.83)	0.03	-	-
DPP4 inhibitor (yes vs. no)	1.77 (0.87–3.59)	0.11	-	-
Metformin (yes vs. no)	1.51 (0.62–3.67)	0.36	-	-
Actos (yes vs. no)	0.17 (0.02–1.51)	0.11	-	-
Insulin (yes vs. no)	12.39 (3.58–45.85)	<0.001	4.67 (1.11–19.59)	0.04
Statin (yes vs. no)	0.98 (0.52–1.86)	0.95	-	-
Laboratory data				
Creatinine, mg/dL	0.83 (0.45, 1.52)	0.55	-	-
Hemoglobin, g/dL	1.16 (0.97, 1.39)	0.09	-	-
Albumin, g/dL	0.35 (0.09, 1.38)	0.13	-	-
Uric acid, mg/dL	0.92 (0.75, 1.11)	0.39	-	-
Cholesterol, mg/dL	1.01 (1.01, 1.02)	0.003	-	-
Log (Triglyceride)	1.01 (1.01, 1.02)	<0.001	1.01 (1.01–1.02)	0.001
HDL, mg/dL	0.99 (0.98, 1.01)	0.49	-	-
LDL, mg/dL	1.01 (0.99–1.01)	0.10	-	-
Glycated hemoglobin, %	2.66 (1.79, 3.95)	<0.001	2.22 (1.36–3.63)	0.001
Lean mass index, kg/m^2^	1.14 (0.80, 1.61)	0.45		
Fat mass index, kg/m^2^	1.43 (0.90, 2.27)	0.12		
Microbiome				
Log (*Firmicutes*/g)	0.81 (0.36, 1.85)	0.62	-	-
Log (*Bacteroidetes*/g)	0.99 (0.59, 1.66)	0.97	-	-
Log (*Firmicutes/Bacteriodetes*)	0.99 (0.96, 1.02)	0.59	-	-
Log (*C. leptum group*/g)	1.01 (0.57, 1.78)	0.96	-	-
Log (*Bacteroides*/g)	1.03 (0.62, 1.72)	0.90	-	-
Log (*Bifidobacterium*/g)	0.99 (0.77, 1.26)	0.95	-	-
Log (*F. prausnitzii*/g)	0.84 (0.62, 1.14)	0.27	-	-
Log (*A. muciniphila*/g)	0.83 (0.72, 0.97)	0.02	0.80 (0.66, 0.99)	0.04
Log (*E. coli*/g)	1.32 (0.95, 1.85)	0.09	-	-

Abbreviations: OR, odds ratio; HOMA-IR, homeostatic model assessment-insulin resistance; BMI, body mass index; HDL, high-density lipoprotein; LDL, low density-lipoprotein.

## Data Availability

The data presented in this study are available on request from the corresponding author. The data are not publicly available due to privacy.

## Supplementary Materials

The following supporting information can be downloaded at: https://www.mdpi.com/article/10.3390/jpm12040617/s1, Table S1: Target sequence of materials utilized in the study. Table S2. The microbiome distribution of study participants stratified by different medications.
