# Supplementary material for: Interrelationship of Gut Microbiota, Obesity, Body Composition and Insulin Resistance in Asians with Type 2 Diabetes Mellitus
Source: J Pers Med. 2022 Apr 11;12(4):617. doi: 10.3390/jpm12040617 (PMC9025506; doi:10.3390/jpm12040617)
Supplement: Supplementary file 1 [file jpm-12-00617-s001.zip › jpm-1651386-supplementary.pdf]

Table S1. Target sequence of materials utilized in the study.

Target	Primer (5'-3')	Product size (bp)	Annealing temp.	Reference
Phylum: *Firmicutes*	F: GGAGYATGTGGTTTAATTCGAAGCA	126	60	Anaerobe 14:224 (2008)
	R: AGCTGACGACAACCATGCAC			
C. leptum group (IV)	F: GTTGACAAAACGGAGGAAGG	245	60	JCM 52:398 (2014)
	R: GACGGGCGGTGTGTACAA			
Faecalibacterium prausnitzii	F: AGATGGCCTCGCGTCCGA	199	60	JCM 52:398 (2014)
	R: CCGAAGACCTTCTTCCTCC			
Phylum: *Bacteroidetes*	F: GGARCATGTGGTTTAATTCGATGAT	126	60	Anaerobe 14:224 (2008)
	R: AGCTGACGACAACCATGCAG			
Bacteroides	F: GTCAGTTGTGAAAGTTTGC	127	60	JCM 52:398 (2014)
	R: CAATCGGGAGTTCTTCGTG			
Phylum: *Actinobacteria*				
Bifidobacterium	F: AGGGTTCGATTCTGCTCAG	156	60	JCM 52:398 (2014)
	R: CATCCGGCATTACCACCC			
Phylum: *Proteobacteria*				
Escherichia coli	F: CATGCCGCGTGTATGAAGAA	96	60	PEDIATRICS118(2):511-21 (2006)
	R: CGGGTAACGTCAATGAGCAAA			
Phylum:*Verrucomicrobia*				
Akkermansia muciniphila	F:CAGCACGTGAAGGTGGGGAC	327	60	Obesity 2257-2261 (2012)
	R:CCTTGCGGTTGGCTTCAGAT			
Total bacteria	F: ACTCCTACGGGAGGCAGCAGT	200	60	JCM 52:398 (2014)
	R: GTATTACCGCGGCTGCTGGCAC			

Table S2. The microbiome distribution of study participates stratified by different medication.

	**Entire Cohort (n=154)**	**SU user (n=71)**	**Non-SU user (n=83)**	**Insulin user (n=29)**	**Non-Insulin user (n=125)**	**Metformin user (n=132)**	**Non-Metformin user (n=22)**	**DPP-4 inhibitor user (n=112)**	**Non-DPP-4 inhibitor user (n=42)**
Firmicutes, copies*10^9/g	4.4(2.3,7.2)	4.4(2.2,6.9)	4.5(2.6,8.2)	**3.2(1.4,5.3)**	**4.8(2.7,8.1)**	4.5(2.5,7.8)	4.1(1.8,6.2)	4.7(2.6,7.0)	3.6(1.7,8.2)
Bacteroidetes, copies*10^9/g	8.9(3.8,17.2)	8.3(3.6,14.6)	9.9(4.3,18.3)	8.6(3.3,15.7)	9.1(3.9,17.5)	8.9(3.8,17.2)	8.1(3.6,17.1)	8.6(3.8,18.2)	10.1(3.7,14.8)
Firmicutes/Bacteroidetes	0.5(0.2,1.1)	0.5(0.3,1.3)	0.5(0.3,1.1)	0.5(0.2,1.0)	0.5(0.3,1.2)	0.5(0.3,1.2)	0.5(0.3,1.0)	0.5(0.3,1.2)	0.5(0.3,1.2)
C. leptum group, copies*10^8/g	5.9(2.2,11.6)	4.8(2.1,9.7)	7.8(2.3,12.2)	4.3(2.3,9.1)	6.4(2.2,12.0)	6.1(2.0,11.4)	5.5(3.5,13.2)	6.5(2.9,11.7)	4.4(0.9,10.8)
F. prausnitzii, copies*10^7/g	11.0(2.0,28.3)	**8.3(0.9,21.4)**	**15.1(3.3,43.8)**	**4.0(2.0,13.0)**	**12.8(2.3,30.6)**	9.6(1.9,28.5)	13.8(5.8,24.6)	11.4(2.4,27.0)	8.1(1.2,34.5)
Bacteroides, copies*10^9/g	1.6(0.8,3.6)	1.4(0.9,3.4)	1.7(0.8,3.9)	1.4(0.8,3.2)	1.6(0.9,3.6)	1.6(0.9,3.6)	1.5(0.8,3.8)	1.6(0.9,3.5)	1.8(0.5,3.8)
Bifidobacterium, copies*10^6/g	3.6(0.3,13.8)	2.6(0.2,9.2)	4.3(0.4,15.7)	**0.7(0.1,5.8)**	**4.4(0.4,14.8)**	3.9(0.3,14.1)	2.7(0.2,10.8)	3.4(0.2,11.7)	4.1(0.5,18.9)
A. muciniphila, copies*10^4/g	0.8(0.2, 410.0)	0.8(0.2,852.2)	0.8(0.2,368.4)	0.8(0.2,2078,0)	0.8(0.2,386.0)	**0.7(0.2,158.5)**	**86.0(0.4,7743.6)**	0.9(0.2,530.7)	0.9(0.4,89.8)
E.coli, copies*10^8/g	1.2(0.3,5.9)	**2.0(0.5,11.7)**	**1.0(0.2,4.5)**	3.3(0.3,17.2)	1.2(0.3,5.8)	1.4(0.3,6.0)	0.9(0.06,6.7)	**1.6(0.3,11.5)**	**0.9(0.3,3.3)**
